# Informing the Design of “Lifestyle Monitoring” Technology for the Detection of Health Deterioration in Long-Term Conditions: A Qualitative Study of People Living With Heart Failure

**DOI:** 10.2196/jmir.6931

**Published:** 2017-06-28

**Authors:** Sarah Hargreaves, Mark S Hawley, Annette Haywood, Pamela M Enderby

**Affiliations:** ^1^ Centre for Assistive Technology and Connected Healthcare School of Health and Related Research University of Sheffield Sheffield United Kingdom; ^2^ Public Health School of Health and Related Research University of Sheffield Sheffield United Kingdom

**Keywords:** independent living, human activities, heart failure, biomedical technology

## Abstract

**Background:**

Health technologies are being developed to help people living at home manage long-term conditions. One such technology is “lifestyle monitoring” (LM), a telecare technology based on the idea that home activities may be monitored unobtrusively via sensors to give an indication of changes in health-state. However, questions remain about LM technology: how home activities change when participants experience differing health-states; and how sensors might capture clinically important changes to inform timely interventions.

**Objective:**

The objective of this paper was to report the findings of a study aimed at identifying changes in activity indicative of important changes in health in people with long-term conditions, particularly changes indicative of exacerbation, by exploring the relationship between home activities and health among people with heart failure (HF). We aimed to add to the knowledge base informing the development of home monitoring technologies designed to detect health deterioration in order to facilitate early intervention and avoid hospital admissions.

**Methods:**

This qualitative study utilized semistructured interviews to explore everyday activities undertaken during the three health-states of HF: normal days, bad days, and exacerbations. Potential recruits were identified by specialist nurses and attendees at an HF support group. The sample was purposively selected to include a range of experience of living with HF.

**Results:**

The sample comprised a total of 20 people with HF aged 50 years and above, and 11 spouses or partners of the individuals with HF. All resided in Northern England. Participant accounts revealed that home activities are in part shaped by the degree of intrusion from HF symptoms. During an exacerbation, participants undertook activities specifically to ease symptoms, and detailed activity changes were identified. Everyday activity was also influenced by a range of factors other than health.

**Conclusions:**

The study highlights the importance of careful development of LM technology to identify changes in activities that occur during clinically important changes in health. These detailed activity changes need to be considered by developers of LM sensors, platforms, and algorithms intended to detect early signs of deterioration. Results suggest that for LM to move forward, sensor set-up should be personalized to individual circumstances and targeted at individual health conditions. LM needs to take account of the uncertainties that arise from placing technology within the home, in order to inform sensor set-up and data interpretation. This targeted approach is likely to yield more clinically meaningful data and address some of the ethical issues of remote monitoring.

## Introduction

### Background

Health services around the world are facing the challenge of providing care for an increasing number of people living with long-term conditions (LTC) within the community [1-3]. The greatest challenges arise from complex or difficult-to-manage conditions—for example, unstable conditions that are subject to exacerbations [4], complex multiple LTCs [5,6], or circumstances that increase vulnerability, such as old age [5] or living alone [7,8]. Health technologies are being developed to help people living at home manage LTCs. One such technology is “Lifestyle Monitoring” (LM), a telecare technology based on the idea that everyday home activities may be indirectly monitored via sensors to give an indication of the changing health state [9-13]. This remote monitoring technology is designed to unobtrusively monitor participants’ home activities over time, with the aim of identifying signs of deterioration as part of the clinical management approaches within a primary or community care setting.

Activity measures are commonly used to assess medical and social needs in older people (eg, [14,15]) by indirectly testing the effects of disease on the ability to perform everyday activities, such as climbing the stairs or preparing a meal [16]. Currently such tests are mostly carried out either by health care professionals observing the performance of activities or by patients self-reporting their abilities. These tests are carried out either on a single occasion or at infrequent intervals. LM technology has been developed to infer home activities on a continuous basis using sensors sited around the home.

Reviews of LM research have highlighted deficits in knowledge [17,18]. Key to this are issues around whether and how home activities change during deteriorations in health. Gokalp et al[18] identified three key areas for further investigation in their review: (1) determining the correlation between changes in activities of daily living and well-being or health status (ie, extracting useful information relevant to subjects’ well-being), (2) determining the most effective set of sensors, and (3) the lack of a decision model which explains how changes in monitored activity relate to changes in health, which in turn could inform the actions of health services. This paper focuses on the first two issues: (1) whether and how home activities change during deteriorations in health, and (2) if changes do occur, how these might be captured by sensors sited around the home. The study also informs debate on the third issue raised by the review, relating to the processes of clinical decision-making using information derived from remote monitoring technologies.

Previous LM studies have described various approaches to remotely monitoring home activities with the aim of detecting deteriorations in health. One common method has been to use sensors to create an electronic version of the activities of daily living (ADL) measure within the home, but this method has so far not provided a practical means of detecting clinically meaningful changes in home activity of patients [17,18]. This study approaches this key issue in a different way. We sought to provide a detailed qualitative exposition of whether and how activities change within the home during the various health states of a long-term condition; and through this lived experience, to inform the design of LM technology.

### Aims

This paper reports the findings of a study examining everyday activities in people with heart failure (HF) in varying health states in order to identify whether participant activities change in distinctive ways across the health states, and particularly during an exacerbation, in an exemplar long-term condition. We sought to capture details of participants’ activities at home at times when they experience these varying health states to inform sensor set-up for remote monitoring and to potentially capture these clinically important changes within the data stream. The study also explored other influences on everyday activity to understand the degree to which activity was influenced by the symptoms of the condition compared to other factors. The research questions were as follows:

1. How do everyday activities undertaken at home by people with HF vary according to the health state of the individual carrying them out?
2. What other factors influence everyday activity in older people?
3. How might changes in home activity be captured indirectly via sensors sited within the home?

All research questions were considered through the lens of LM, the remote monitoring technology, and from the perspective of the lived experience of the long-term condition. The focus of the study was HF, but the aims of the study were broader: to add to the to the knowledge base informing the development of home monitoring technologies designed to detect health deterioration in any long-term condition that is subject to exacerbations.

Within the United Kingdom, approximately 493,000 people are recorded on general practice registers of patients with HF [19]; although, the actual prevalence is likely to be higher. HF is a complex condition with an uncertain trajectory [20-23] and a high symptom burden [24]. The symptoms are known to impact everyday activities (eg, [25-29]) in ways that vary depending on the degree of intrusion from symptoms. For example, Edmonds et al [26] documented changes in activity in response to different degrees of breathlessness. The condition is costly both to the individual and to health services because of the risk of non-elective hospital admission and readmission [30]. It is known that people with HF may delay seeking help during an exacerbation, and therefore, symptoms may deteriorate before health services are alerted [31-33]. One aspect of this delay may arise from difficulties with symptom recognition [32-37]. Early detection of worsening symptoms may potentially halt an exacerbation, allowing the condition to be stabilized via a community health care intervention, thus avoiding the need for hospital admission. This research, therefore, particularly focused on the potential changes in activity leading up to an exacerbation and how these can be detected by sensors.

## Methods

### Participants and Recruitment

The study participants with HF were recruited in two ways. Participants were either identified by a heart failure specialist nurse and recruited by the research team, or were recruited from a HF support group. All participants lived in and around Barnsley, a town in the north of England.

The sample was chosen to include a diverse experience of living with HF, and in particular, participants varied in their age, severity of HF, and the length of time since diagnosis (Table 1). All participants were aged 50 years and above. Additional insights were gained from the partners of some of the participants with HF, who, at the participants’ request, sat in and contributed to the interview.

### Study Design

Participants were interviewed in their own homes over an 8-month period using a semistructured schedule both to ensure that key topics were discussed and to capture a broad range of experience. The key topics were identified by literature searching and included a focus on HF symptoms and activities undertaken during days classified as normal days, bad days, and exacerbations [38,39,25].

The audio-recorded interviews were typically of one hour duration, with a minimum of 40 minutes and a maximum of two hours. After each interview, field notes were taken to both reflect on the interview and gain insights into the research questions—these notes provided the starting point for data analysis. The interviews were transcribed in full.

### Ethics

Ethical approval was gained from Leeds (East) Research Ethics Committee (08/H1306/46) and research governance from Barnsley Hospital NHS Foundation Trust. Participants were sent written information about the project at the initial point of recruitment. Key points were reiterated before the interview and participants were informed of their right to stop the interview at any time. Written consent was then taken.

### Theoretical Framework and Analytical Approach

The project was underpinned by subtle realism: an epistemological stance based on the belief that there are multiple non-contradictory versions of reality and that although accounts differ, they all reflect reality [40]. Researchers sought to cast a critical eye over participants’ accounts of life with HF to find a version “...of whose validity we (were) reasonably confident” ([41], page 50). Researchers were mindful of their own influence on this process and sought, through reflexivity, to identify any erroneous assumptions, attitudes, or beliefs in a process described by O’Cathain [42] as “situating” yourself within the research.

The interview data were analyzed using template analysis [43-45], a form of thematic analysis where the key tool is the use of a hierarchical template to summarize themes and provide a visual aid to both analysis and data interpretation. The hierarchical structure of the template is used to organize themes in a meaningful way, with key broad concepts captured in higher level themes, and lower level themes providing more detail to improve understanding of specific aspects of the broader themes.

### Data Analysis

The data were coded by Sarah Hargreaves, with checks made by other researchers to validate choice of codes. A minority of initial themes were informed by the literature (a priori themes); however, these were retained only if the theme was found to be both present within the data and of relevance to the research question. If not, a priori themes were amended to reflect the data or discarded if they were found to be no longer appropriate. Other themes were identified from “features” of participant accounts (perceptions or experiences) relevant to the research questions. Initial theoretical propositions were tested at a validation exercise undertaken at a heart failure support group meeting with 27 attendees. Themes were further consolidated via the process of template development whereby different template structures were explored in order to reach a final version that both represented the themes within the interviews and directly addressed the research questions. Consider, for example, the high level theme “Deciding what to do today,” which arose out of an understanding from the interviews that activities on any given day are influenced by a wide range of factors. These were detailed in lower-level codes, such as the weather, commitments, and activity assessment. The understanding of this theme was consolidated by the validation exercise, which highlighted not only the importance of symptoms as a key influence on daily activity but also individual differences. This process of template development was documented in order to create an audit trail [45].

The data were initially coded manually and at a later stage imported into NVivo 9 (QSR International) in order to utilize the text retrieval facilities to aid template development and for reporting purposes.

In this paper, all participants are referred to by pseudonyms.

## Results

### Participant Characteristics

Table 1 summarizes the characteristics of the interview participants with HF. Participants ranged in age, from younger participants in their 50s to older participants in their 80s, with the majority in their 70s. In addition, many participants suffered from a range of co-morbidities from mild conditions (such as mild arthritis) to potentially life-threatening ones (eg, cancer). The sample included participants who had been diagnosed with HF recently (within the last three years), and those who had been living with HF for longer. Despite efforts to recruit a gender-balanced sample, the sample was predominantly male.

The partners or spouses of 11 interviewees sat in and contributed to the interviews.

**Table 1:** Characteristics of participants with heart failure.

NYHA heart failure class^a^	Gender	Length of time since diagnosis^b^		Grand total
		Within the last 3 years	>3 years	
I	Male	1		1
II	Female	1		1
	Male	5	1	6
III	Female	1	1	2
	Male	1	6	7
IV	Female	1		1
	Male	1	1	2
Grand total		11	9	20

^a^Classified according to participant accounts of the impact of symptoms on everyday activity and interviewer observations [38].

^b^This information was derived from participant accounts.

During the analysis an integrative theme was identified [44]. This is a theme which King describes as “undercurrents running through participants’ accounts” (page 460), and is thus pertinent to the interpretation of much of the data. In this study the integrative theme was the degree of intrusion from symptoms.

### The Three Health States of Heart Failure and Impacts on Everyday Activity

The interviews confirmed that participants recognized the concept of normal and bad days and were able to describe how these two types of days differed both in terms of the degree and type of symptoms experienced, and the sort of activities that they would be likely to undertake. A normal or good day was relatively symptom-free, and this gave participants more energy to undertake everyday activities, which might be within the home. For some, this feeling of well-being inspired participants to undertake activities in the garden or to go out.

Well on a good day I live like a normal day virtually, don’t I?...Erm if I want to go for a walk, we go for a walk. We go shopping, whatever, visit people.

Brian

How often these good times occurred, how long they lasted, and the degree of extra energy experienced, all varied depending on the severity of HF. For some, these good times were both infrequent and transient.

In contrast bad days were characterized by a lack of energy (“a whole system tiredness”), a lethargy, and with it an accompanying feeling of listlessness, irritation, and negativity. During such times participants preferred to stay at home and may have undertaken gentle activities or those requiring very little energy, such as watching the television or using the computer. Again the degree of intrusion from the symptoms varied, with some participants very badly affected by bad days and unable do anything, while others ignored their symptoms and kept going.

You can have days when you feel really down and you know it just seems like you are useless, you can’t do anything and you don’t have the energy to get a meal or anything

Dawn

Bad days were regarded as a transitional state to be endured in order to reach better times.

The third health state was exacerbation. Not all the participants had experienced an exacerbation, but for those who had, it was remembered with horror—as a time when health concerns came to the fore and everything became a struggle. The key symptoms were breathlessness, tiredness, and swelling (oedema), with breathlessness especially reaching frightening levels as participants described struggling for breath.

...Just if you were walking up a little incline erm that was something or nowt [nothing] really you had to stop and you would be going [participant acts out doing heavy gasping breaths, followed by a deep lurching breath] and feel as if you couldn’t get enough in.

Larry

Exacerbations differed from bad days because of the degree of exercise intolerance and dyspnoea, and the perception that the body was out of control, trapped in a “downward spiral.”

The impact on home activities built up over time, with participants describing how they spent more time at home, and even there, were limited and selective in their activities. A notable aspect of activity within this health state was slowing down, when activities were undertaken very slowly, especially more exerting activities, such as walking and climbing the stairs.

Erm but just everything was just so slow, so very, very slow.

Willow

The exacerbation also changed activities at night, with sleep broken by episodes of breathlessness which prompted the participants to undertake activities to ease their breathing, such as sitting on the edge of the bed or going downstairs to sit the night out on the sofa.

I come down and was watching sport two, three, four o’clock in the morning, and was dozing on the settee. You know awful, awful feeling when you waken in the night-time like that.

Larry

A number of participants spontaneously described how the first thing that alerted them to the onset of exacerbation was a new or increased difficulty in walking or using the stairs.

I had been having this increasing breathlessness and then the snow, I couldn’t go further than five yards with a bag and I had to stop myself and think this is ridiculous.

Janice

It was walking really. I had always been a fast walker and I had to slow down.

Willow

During an exacerbation of symptoms, walking became an ordeal as the physical effort caused breathlessness. The length of an acceptable walk was scaled down, and the activity was carried out more slowly than normal with stops to breathe. This change was observed both inside the home and outside.

...from the top of the stairs into the toilet I was absolutely shattered. From there to the chair I was shattered, from that chair to the end of the settee there [a few steps] and I would have to stand [sound of panting] out of breath completely.

Eric

...Just walking to the bottom of the street really slowly and if other people saw me that know me, you should have seen the look on their faces to say what’s the matter with you?

Larry

As the exacerbation progressed, participants described how activity became pared down to absolute necessities and they avoided climbing the stairs as it became too exhausting and distressing.

### Other Factors Influencing Activity

The interviews explored the broad experience of living with HF, and the accounts highlighted how participants were chiefly concerned with living their lives in spite of HF.

Err...I don’t see myself as being bad, poorly, ill. Whatever you want the classification to be. I don’t. Well it is just me, I try not to see that part, I think I am up and running, I am okay.

Alistair

This stoical attitude presents a challenge to the concept of LM because individuals with HF may not necessarily respond to intrusive symptoms by changing activity. The most extreme example of this came from a participant who, while suffering an exacerbation, ignored his symptoms and attempted to carry on.

I always remember that the Doctor said to me…he said you are not working Mr Smith are you? Well I have been to the gym this morning, I have been to work all day, I just had a shower and I have run down here. He said you could have collapsed and died, this was the cardiologist.

Larry

Participant accounts gave an understanding of how everyday activity is influenced by both the individual situation of the participant (eg, severity of HF and co-morbidities, age, personality, gender, lifestyle, attitude to activity), and external factors (eg, housing, local geography, transport availability, weather, demand on time) A good example of this came from Janice, a participant juggling the demands of bringing up her children while living with HF.

What I think I should do is put an Agatha Christie on the DVD, have someone bring you afternoon tea and put your feet up…But life’s not like that.

Janice

The interviews illustrate that in real life, the relationship between activity and health is not clear cut, thus LM systems would need to take account of this complexity and individual circumstances.

### Potential Targets for Activity Monitoring to Detect Signs of Exacerbation

Participant accounts verified that an exacerbation is a distinctively different health state, and they also identified activities that were undertaken in order to ease symptoms. Participants described how symptoms built up over time during the exacerbation, and often they did not seek medical help until the symptoms were intolerable —for some in spite of their close family urging them to seek help.

The accounts of the experience of activities undertaken during different health states could provide the starting point for developing a platform of LM sensors to monitor home activities linked to an exacerbation of symptoms. Table 2 lists activities which could potentially be monitored as well as insights into how these changes might be monitored. During an exacerbation of symptoms, participants with HF, for example, described both changes to existing activities (eg, reduced speed in walking and climbing the stairs, staying at home more, and being more sedentary) and undertaking new activities in response to distressing symptoms (eg, sitting up at night to aid breathing, perhaps watching television).

The interviews also highlighted the importance of signs of ill-health relating to appearance, which would require new sensors to be developed. An important sign was swelling, with participants describing the extent of swelling to their body during an exacerbation, as described by Willow:

My legs were very swollen, if I sat down I needed to get them up and Edward used to have to lift them up because they were just so heavy, I couldn’t lift them up.

This swelling was reported in feet, legs, the abdomen, and even fingers. More subtle signs noted by the partners of participants with HF were changes in facial appearance including expression, appearance of eyes, blue lips, and alteration in mood and demeanor. In addition, during ill-health, the role of partners changed and they undertook more activities to support their spouse.

**Table 2:** Activities that change during an exacerbation and insights into how these changes might be monitored.

Home activity	Details of activity changes taking place within the home when health declines	How these changes might be monitored
Climbing the stairs	Stairs became more of a physical challenge during an exacerbation, and the climb is likely to be undertaken more slowly (the individual may also rest during the climb), and over time the activity is likely to be undertaken less frequently than usual or avoided, as symptoms spiral out of control.	A means of counting both the number and duration of stair ascents and descents should be sought—potentially with a device recording the time spent climbing from the first to the last step.
Dressing	Participants were aware of shoes or clothes becoming tighter. Dressing becomes more of a physical effort, especially putting on socks.	Some means of reporting that clothes or shoes have become tighter would be a useful indicator of increased fluid build-up. It would be important to find out the order in which parts of the body become swollen, as the fluids build up; the normal pattern in individuals should also be established.
Food preparation	Food preparation is simplified or avoided.	Information about normal food preparation methods would need to be sought in order to monitor and measure the duration of these tasks, as during periods of ill-health participants described shorter and less elaborate cooking methods. Microwave usage may potentially increase during periods of ill-health, in order to reheat previously prepared meals, or shop-bought ready meals; this could be measured using an electrical socket device.
Staying at Home	A reduction in leaving the home, and then cessation. Participants withdrew from social activities as their health declined.	A key sensor could be used to monitor when the door used to go out is locked from outside (this may differ from the door used to access the garden). The key sensor could provide an indirect means of measuring social isolation, as people may cancel social activities. A means of reporting the scale of breathlessness during speech could provide an indicator of worsening breathlessness.
Increase in sedentary activities	An increase in sedentary activities in the home, such as sitting on the sofa; as health declines, chores are likely to be limited to necessities, and activities requiring physical exertion avoided	See “sleeping on the chair/sofa” below
Activities undertaken to ease breathlessness	Some people seek air during episodes of breathlessness, opening windows or doors, using a fan, or sitting upright.	Windows and doors opening and closing could be monitored with a door sensor, with openings that occur at night potentially being of greater significance. Window monitoring would only be of value if the window was generally closed.
Sleeping in the bed	Nocturnal sleep may be broken by episodes of breathlessness, requiring the participant to sit upright, or sit on the edge of the bed to recover breath	Mattress monitors are currently used to measuring bed occupancy, although they would not be able to detect subtle signs of ill-health, such as more restless sleep. A means of monitoring the duration of time spent sitting on the edge of the bed is needed.
Sleeping on the Chair/sofa	When breathlessness is bad, some people resort to sleeping upright in a chair, or they may go down stairs in the night to sit out the night on the sofa	Information about where people sleep when they feel unwell should be sought in order to monitor use of this alternative sleeping place, as participants commonly mentioned sleeping on the sofa or chair in the lounge during episodes of nocturnal breathlessness. This measure of chair usage would be more reliable if the chair was used exclusively by the participant; and this is not unrealistic, since some participants had an upright chair that they favored.
Signs noted by partners: Symptoms and Subtle signs	Partners were aware of a deterioration of symptoms, such as increased breathlessness, and swelling, and began to help their partners undertake activities. People talk less during a decline of health, and withdraw into self. A change of appearance of the face, eyes, and demeanour was noted by partners during an exacerbation.	Some means should be provided for partners/family to report observations about the appearance of their relative with a long-term condition. Partners were very in tune with their partner’s health, and thus this valuable information should be fed into the monitoring.
Television viewing	Viewing may increase during an exacerbation, with an increase in viewing during the day, and viewing at unusual times (such as in the night) may be a particular indicator	Information about television viewing would need to be sought before monitoring, as people may always have the television on as background noise, or undertake other activities during periods of ill-health—for example, listening to the radio more. A television electrical socket device could record the duration of time the television is on, but not whether it is actually being watched. A measure of night-time viewing may be an important indicator of a change in health.
Movement around the home	Walking becomes more of a physical challenge during an exacerbation, and is undertaken more slowly and less frequently.	The number of daily steps could be recorded via a pedometer, and speed recorded via an accelerometer

## Discussion

### Principal Findings

The basis of LM technology is that the home activities of people at risk of an exacerbation of a long-term condition may be monitored via sensors in order to provide an indication of important changes in health. This study found that a sensor platform could potentially be tailored to identify signs of an exacerbation in HF when the setup is informed by understanding the lived experience of HF, and in particular the impact of intrusion of HF symptoms on everyday activity. We argue that this emphasis on understanding the lived experience of long-term conditions is important both for identifying which health conditions may be appropriate for LM and for tailoring the sensor setup to identify clinically important changes in health. While this study focuses on HF, the approach has a far wider application for the development of approaches to remote monitoring and data analysis in long-term conditions.

The findings suggest the need for a more personalized and targeted approach to LM to tailor sensor setup to monitor activities which are potential indicators of important changes in health. By exploring home activities during the different health states of HF, we were able to identify activities which should be monitored, and from this, choices could be made about identifying appropriate means of detecting change. This requirement to identify specific activities that are sensitive to changes in symptoms (specific to a health condition) has not been addressed by the LM literature, where descriptions of systems, methods, and the context in which they are being used, tend to be vague [17].

Although it was known that people with health conditions change their behavior during an exacerbation (eg, during an exacerbation people with HF may sleep upright), detailed description of how these changes occur within the home were lacking [46]. A key finding from this study is the detailed list of changes that occurred during exacerbations (Table 2) and this offers useful information for the developers of sensors and platforms, and to inform the development of algorithms to detect changes in the data collected. This key finding adds to insights gleaned from other studies looking at varied long-term conditions—Clarke, for example [46].

This targeted approach offers the potential for a reduction in the number of sensors targeted at specific activities, which may provide more clinically meaningful data than a standard broad-brush setup [47]. It may also lead to the development of sensors designed specifically to monitor activities of clinical significance. LM has been criticized for the failure to capture clinically significant data, and for Elwyn et al [48], this arises from the focus on data that is easy to collect rather than data that is indicative of a change in health. It is anticipated that LM data could inform clinical management of patients living within the community, offering a potential safety net, so that primary care services could be alerted to signs of decline in order to intervene early and potentially prevent hospital admission. In our study, participants delayed seeking help for an exacerbation, a finding in keeping with other HF studies [31-33]. A LM service may be of great value to patients, given that it would require no effort from the patient, and may reduce some of the uncertainty and complexity of living with unpredictable conditions.

An approach focused on collecting data of clinical significance is more ethical, as it is more likely to yield data of value to health professional decision making, leading to interventions of clinical benefit [17,48]. In addition, the creation of a bespoke LM setup for a specific health condition, which also meets the needs of users, is more likely to yield positive benefit for the user and inspire confidence in health professionals. This is a vital step in the wider adoption of LM [49].

It is likely that LM is more appropriate for some health conditions than others. This study identified some aspects of HF that make it more appropriate for LM. First, the health states within the condition were found to be distinctly different, and in particular there were specific activities found to be undertaken during an exacerbation to ease symptoms, as well as broader changes in activity that occurred (such as staying at home more) during exacerbations. In addition, the exacerbation was found to build up over time, and this would allow sufficient time to identify a change in health and intervene. Participants of the study were able to identify the first signs of an exacerbation, and this could inform the sensor setup. Although taking a bespoke approach to LM design should make it applicable to other long-term conditions, the characteristics of other conditions may make a LM approach less likely to be successful. The buildup to an exacerbation in chronic obstructive pulmonary disease (COPD) is much faster (eg, [50]). A COPD telecare study was unsuccessful in identifying the early stages of an exacerbation due to a lack of early predictors [51], and the authors speculated that the system may have been identifying signs of a bad day rather than an exacerbation.

Another potential means of enhancing the remote monitoring would be to feed in additional information. The study found that the partners of people with HF were undertaking an informal monitoring role, that is, observing their partner day and night for signs and symptoms that their health was deteriorating. This monitoring included aspects which current sensors would not be able to capture, such as observing changes in the appearance of their partner, their symptoms (observing increased breathlessness, for example), mood, and noting additional activities they were required to undertake. Other studies have noted this monitoring role as well [25]. Such observations could potentially be clinically significant, or highlight a need for closer examination by a health professional [52]. A means of feeding in this information would add an extra valuable dimension to the resources for decision making [53]. There are, however, ethical implications and potential negative impacts on family relationships arising from this approach which would need further exploration.

Remote monitoring of HF typically focuses on physiological changes [54]. Utilizing technologies that measure physiological changes in parallel with LM provides opportunity to assess the respective accuracy of both data sources and adds an additional depth of information [47]. It would also provide an additional safety net for patients. The first change in activity noted by participants of this study during an exacerbation was a slowing of their walking pace or in climbing the stairs, but they were also aware of increased breathlessness. Unlike physiological measurement technologies, LM technologies require no active engagement from those being monitored, and thus may detect exacerbations in those people unlikely to actively engage with monitoring. The continuous nature of LM may also lead to earlier detection of the beginning of an exacerbation. Future research might focus on how measures of physiological change and activity vary across the timeline of an exacerbation within the complexity of the lived experience of HF.

Complexity arises from placing technology within the home, where the monitoring interacts with the individual circumstances in which a person lives and the external factors impacting on everyday life. The health state exists not as a separate entity, which directs home activity, but within a complex environment, where factors interact and there may be conflicting demands. This presents a challenge to LM because an individual may feel ill but not attend to those feelings by resting or staying at home, as their lifestyle has other demands. It is well known that people do not respond uniformly to the challenges of ill health [55-58]; and that people with health issues may prefer to focus on living their lives, rather than the health condition [55,58,59]. This means that there will be a level of uncertainty in the data recorded by LM sensors. However we argue that by understanding more about an individual and their attitudes, priorities, circumstances, and routine, this would provide a context within which to both tailor sensor setup to fit in with individual circumstances and behaviors, and also a means of interpreting the LM data in order to reduce the margin of error [60]. We argue that this understanding is important in order to establish a link between the sensor data and the person being monitored. This would require a degree of personalization of sensor setup and interpretation, which would involve additional costs [61]; it may also involve the development of new sensors. This requirement for technologies to be designed “in the wild,” taking account of how real people live in their homes is an important element in designing technologies to fit needs [62].

### Limitations

The participants recruited to this study were varied in terms of age and HF severity, and therefore may not be representative of the typical elderly HF patient cared for by general practice [63]. This study focused entirely on HF, but for some the situation is complicated by co-morbidities [30], and the presence of these is likely to make LM more challenging. The small sample size is a limitation of the study, as it is unlikely that we have captured all aspects of behavior within the home in individuals with HF.

There was an imbalance within the sample, with a greater proportion of male participants and few participants who lived alone. In addition, the study participants were all from a similar geographical area within Northern England and were from the same ethnic grouping (white British), and this may have in part influenced the way that participants responded to the challenges of living with a long-term condition. Certainly participants were stoical in their approach to living with HF, which may reflect the culture of an area with a history of coal-mining and steel-working. This stoical approach to living with a long term condition has been noted by another study based in Northern England [64].

It is acknowledged that there is a risk that the presence of partners may have influenced the interviews (eg, [65-67]). However, we argue that the presence of partners was positive as they were able to provide vivid descriptions of the impact of HF on activity and to act as a counterbalance to the instinct of many of the participants with HF to underplay symptoms.

### Conclusions

This study shows the importance of understanding of the lived experience of long-term conditions in order to potentially identify which conditions are suitable for LM and of detailed information about how home activities change when health deteriorates. This detailed information is the key to designing a sensor setup tailored to both specific health conditions and individual circumstances and to inform data analysis and interpretation. By this means it is anticipated that LM can provide timely, clinically meaningful data that will contribute to the management of long-term conditions of people living in the community.

**Acknowledgments**

The Authors wish to thank Professor Nigel King from the University of Huddersfield for his expert advice on template analysis. Dr Hargreaves was funded by Barnsley Hospital NHS Foundation Trust.

## Abbreviations

ADL: activities of daily living
COPD: chronic obstructive pulmonary disease
HF: heart failure
LM: lifestyle monitoring
LTC: long-term conditions
